# Supplementary material for: Global, regional, and national survey on burden and Quality of Care Index (QCI) of orofacial clefts: Global burden of disease systematic analysis 1990–2019
Source: PLoS One. 2025 Jan 8;20(1):e0317267. doi: 10.1371/journal.pone.0317267 (PMC11709310; doi:10.1371/journal.pone.0317267)
Supplement: S1 Appendix — (PDF) [file pone.0317267.s001.pdf]

QCI of Orofacial Clefts - analysis

Ahmad Sofi-Mahmudi

2024-03-24

Loading required packages

```
pacman::p_load(dplyr,
               ggplot2,
               knitr,
               tidyr,
               readstata13,
               here,
               gt)
```

Loading the dataset

```
main_data = read.dta13(here("data", "main_data.dta"))
iso3 = read.dta13(here("data", "ISO3.dta"))
```

Results

Global Quality of Care (QCI):

```
kable(main_data %>%
        select(location_name, year, sex_name, pca_score) %>%
        filter(location_name == "Global",
                      year == c(1990, 2019)) %>%
        mutate(across(4, round, 1)))
```

```
## Warning: There was 1 warning in `mutate()`.
## i In argument: `across(4, round, 1)`.
## Caused by warning:
## ! The `...` argument of `across()` is deprecated as of dplyr 1.1.0.
## Supply arguments directly to `.fns` through an anonymous function instead.
##
##   # Previously
##   across(a:b, mean, na.rm = TRUE)
##
##   # Now
##   across(a:b, \(x) mean(x, na.rm = TRUE))
```

location_name	year	sex_name	pca_score
Global	1990	Both	66.4
Global	2019	Both	90.2

location_name	year	sex_name	pca_score
Global	1990	Female	82.9
Global	2019	Female	94.3
Global	1990	Male	72.8
Global	2019	Male	93.6

And its plot:

```r
main_data %>%
        filter(location_name == "Global" & age_name == "Age-standardized") %>%
        ggplot(aes(year, pca_score, group = sex_name, color = sex_name)) +
        geom_line(size = 1, alpha = 0.8) +
        geom_point(size = 2) +
        scale_color_brewer(name = "Sex", palette = "Set1") +
        xlab("Year") +
        ylab("Quality of Care Index (QCI)")
```

```
## Warning: Using `size` aesthetic for lines was deprecated in ggplot2 3.4.0.
## i Please use `linewidth` instead.
## This warning is displayed once every 8 hours.
## Call `lifecycle::last_lifecycle_warnings()` to see where this warning was
## generated.
```

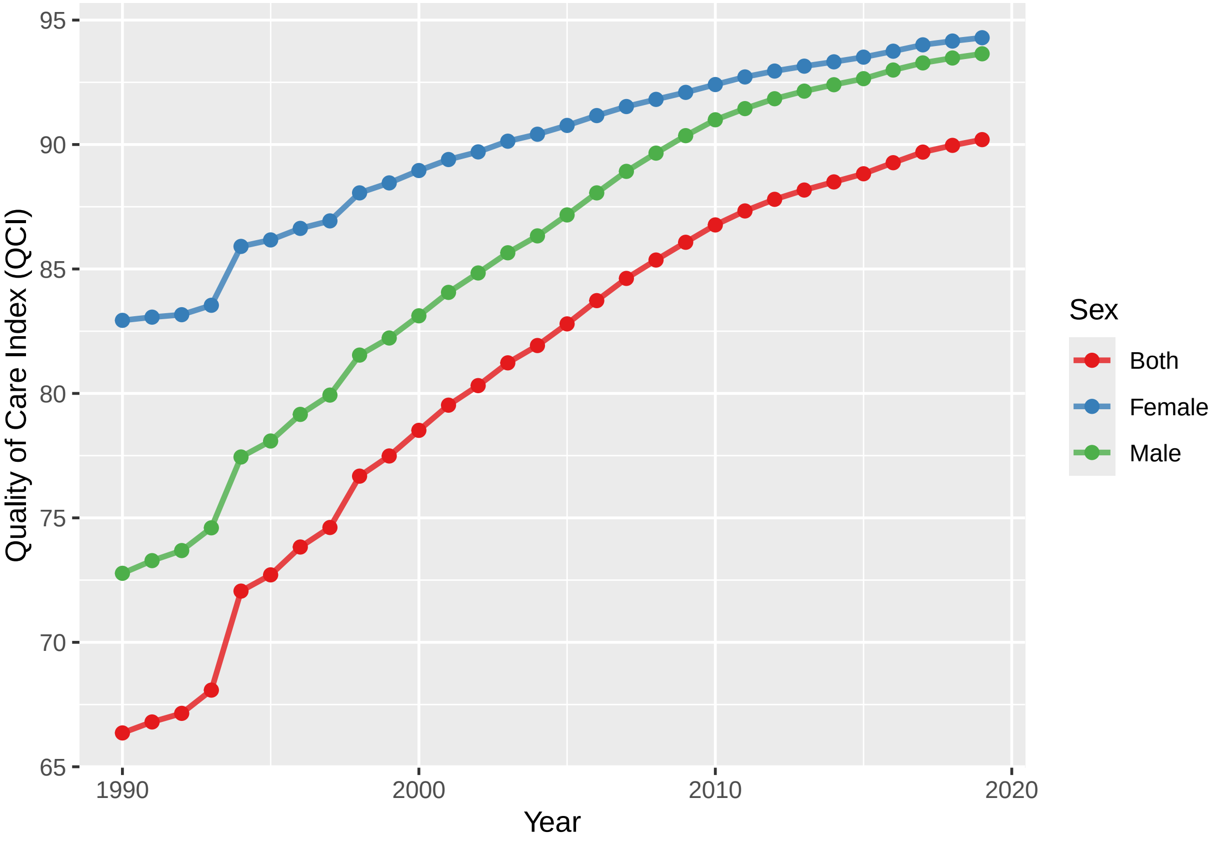


```r
# dev.copy(tiff, "figures/Figure1.tiff", width = 20, height = 10, units = "cm", res = 300)
# dev.off()
```

Top countries:

```
kable(head(main_data %>%
        filter(year == 2019 & sex_name == "Both") %>%
        select(location_name, pca_score)  %>%
        arrange(desc(pca_score)) %>%
        mutate(across(2, round, 1))
     ))
```

location_name	pca_score
Norway	99.9
Ireland	99.4
France	99.4
Germany	99.3
Netherlands	99.3
Malta	99.3

Countries with the least QCI:

```
kable(tail(main_data %>%
        filter(year == 2019 & sex_name == "Both") %>%
        select(location_name, pca_score)  %>%
        arrange(desc(pca_score)) %>%
        mutate(across(2, round, 1))
     ))
```

	location_name	pca_score
217	Sierra Leone	75.3
218	Mali	74.4
219	Ethiopia	73.0
220	Burkina Faso	72.6
221	Niger	67.6
222	Somalia	59.1

Trend of change in QCI:

```
qci_trend = merge(main_data, iso3, by = "location_id") %>%
        select(location_name.y, year, sex_name, pca_score, type, iso3_countries) %>%
        filter(year %in% c(1990, 2019)) %>%
        distinct() %>%
        spread(year, pca_score) %>%
        mutate(year_ratio = `2019`/`1990`,
               compare = ifelse(`2019` > `1990`, "higher", "lower")) %>%
        mutate(year_ratio_cat = ifelse(year_ratio < 0.95, "lower",
                                       ifelse(year_ratio > 1.05, "higher", "equal")))
```

Countries in which the QCI has decreased:

```
kable(qci_trend %>%
        filter(sex_name == "Both" & type == "Country" & compare == "lower"))
```

location_name.y	sex_name	type	iso3_countries	1990	2019	year_ratio	compare	year_ratio_cat
Albania	Both	Country	ALB	98.07684	97.30689	0.9921495	lower	equal

location_name.y	sex_name	type	iso3_countries	1990	2019	year_ratio	compare	year_ratio_cat
Guam	Both	Country	GUM	96.41681	96.15187	0.9972522	lower	equal
New Zealand	Both	Country	NZL	99.06335	98.84734	0.9978196	lower	equal
Northern Mariana Islands	Both	Country	MNP	96.36154	96.27993	0.9991530	lower	equal
Norway	Both	Country	NOR	100.00000	99.90309	0.9990309	lower	equal
Singapore	Both	Country	SGP	99.23507	99.19500	0.9995961	lower	equal
Somalia	Both	Country	SOM	59.34079	59.05250	0.9951418	lower	equal
Zimbabwe	Both	Country	ZWE	85.88404	81.13136	0.9446616	lower	lower

```
qci_trend$type2 = factor(qci_trend$type, levels = c("Global", "WHO Regions", "World Bank Income Levels"

rows = (seq(nrow(filter(qci_trend, sex_name == "Both")))-1) %/% 37

qci_trend %>%
        dplyr::rename(Scale = type2, Location = location_name.y, QCI = `2019`) %>%
        filter(sex_name == "Both") %>%
        select(Scale, Location, QCI) %>%
        arrange(Scale, desc(QCI)) %>%
        mutate(QCI = round(QCI, 1)) %>%
        split(rows) %>%
        do.call(cbind, .) %>%
        #dplyr::rename(Scale = c(1, 4, 7, 10, 13), Location = c(2, 5, 8, 11, 14), QCI = c(3, 6, 9, 12,
        gt() %>%
          cols_label(
                ends_with("QCI") ~ "QCI",
                ends_with("Scale") ~ "Scale",
                ends_with("Location") ~ "Location") %>%
        data_color(
                columns = c(`0.QCI`, `1.QCI`, `2.QCI`, `3.QCI`, `4.QCI`, `5.QCI`),
                method = "numeric",
                palette = c("red", "green"),
                domain = c(59.1, 99.9)) %>%
        data_color(
                columns = c(`0.Scale`, `1.Scale`, `2.Scale`, `3.Scale`, `4.Scale`, `5.Scale`),
                method = "factor",
                palette = "Set2"
        ) %>%
        gtsave("fig.png")


qci_trend %>%
        filter(sex_name == "Both") %>%
        select(location_name.y, type2, `2019`) %>%
        arrange(type2, desc(`2019`)) #%>%
```

```
##                    location_name.y                     type2     2019
## 1                           Global                    Global 90.19779
## 2                  European Region               WHO Regions 97.11111
## 3           Region of the Americas               WHO Regions 93.38609
## 4       Eastern Mediterranean Region               WHO Regions 92.02133
## 5            South-East Asia Region               WHO Regions 91.62646
## 6            Western Pacific Region               WHO Regions 91.29025
```

```
## 7                       African Region            WHO Regions 82.94872
## 8              World Bank High Income World Bank Income Levels 98.68135
## 9      World Bank Upper Middle Income World Bank Income Levels 92.88296
## 10     World Bank Lower Middle Income World Bank Income Levels 91.16962
## 11              World Bank Low Income World Bank Income Levels 80.33064
## 12                           High SDI                      SDI 98.51045
## 13                    High-middle SDI                      SDI 93.84630
## 14                         Middle SDI                      SDI 93.03736
## 15                     Low-middle SDI                      SDI 90.98462
## 16                            Low SDI                      SDI 84.72268
## 17                             Europe          4 World Regions 96.87435
## 18                            America          4 World Regions 93.38609
## 19                               Asia          4 World Regions 91.74093
## 20                             Africa          4 World Regions 84.87962
## 21                      North America       World Bank Regions 98.58294
## 22        Europe & Central Asia - WB       World Bank Regions 97.08795
## 23   Middle East & North Africa - WB       World Bank Regions 95.66187
## 24    Latin America & Caribbean - WB       World Bank Regions 92.37971
## 25                  South Asia - WB       World Bank Regions 91.82220
## 26           East Asia & Pacific - WB       World Bank Regions 90.30872
## 27            Sub-Saharan Africa - WB       World Bank Regions 82.38047
## 28                             Norway                  Country 99.90309
## 29                            Ireland                  Country 99.37874
## 30                             France                  Country 99.35528
## 31                            Germany                  Country 99.34695
## 32                        Netherlands                  Country 99.33693
## 33                              Malta                  Country 99.33319
## 34                              Spain                  Country 99.33005
## 35                           Portugal                  Country 99.32887
## 36                            Uruguay                  Country 99.31392
## 37                             Israel                  Country 99.29824
## 38                              Chile                  Country 99.28937
## 39                              Italy                  Country 99.27780
## 40                             Canada                  Country 99.26611
## 41                            Denmark                  Country 99.26362
## 42                            Austria                  Country 99.26275
## 43                            Iceland                  Country 99.25965
## 44                         Luxembourg                  Country 99.25373
## 45                          Australia                  Country 99.25336
## 46                            Belgium                  Country 99.24154
## 47                            Andorra                  Country 99.20224
## 48                             Sweden                  Country 99.19927
## 49                          Singapore                  Country 99.19500
## 50                     United Kingdom                  Country 99.15592
## 51                             Greece                  Country 99.15589
## 52                           Slovenia                  Country 99.12773
## 53                            Hungary                  Country 99.09388
## 54                          Argentina                  Country 99.08915
## 55                     Czech Republic                  Country 99.05188
## 56                            Croatia                  Country 99.03882
## 57                           Bulgaria                  Country 99.02584
## 58                          Lithuania                  Country 98.98025
## 59                             Brunei                  Country 98.97715
## 60                             Latvia                  Country 98.96509
```

```
## 61                South Korea                  Country 98.92488
## 62                     Panama                  Country 98.89643
## 63                    Romania                  Country 98.87222
## 64                New Zealand                  Country 98.84734
## 65                 Costa Rica                  Country 98.83433
## 66                    Moldova                  Country 98.82697
## 67                     Cyprus                  Country 98.82615
## 68                      Japan                  Country 98.69619
## 69                     Poland                  Country 98.65582
## 70                     Serbia                  Country 98.62487
## 71              United States                  Country 98.50286
## 72                Puerto Rico                  Country 98.41362
## 73                    Bermuda                  Country 98.39093
## 74                 Montenegro                  Country 98.35171
## 75                   Slovakia                  Country 98.34010
## 76                The Bahamas                  Country 98.33795
## 77        Trinidad and Tobago                  Country 98.32139
## 78                       Cuba                  Country 98.31545
## 79                   Mongolia                  Country 98.30684
## 80                 Uzbekistan                  Country 98.29204
## 81     Bosnia and Herzegovina                  Country 98.25654
## 82                    Belarus                  Country 98.19649
## 83                    Finland                  Country 98.14070
## 84                 Kazakhstan                  Country 98.09598
## 85        Virgin Islands, U.S.                 Country 98.09018
## 86                 Azerbaijan                  Country 98.08890
## 87                    Georgia                  Country 98.00003
## 88                    Grenada                  Country 97.98003
## 89                   Colombia                  Country 97.95313
## 90                 Kyrgyzstan                  Country 97.92165
## 91                  Macedonia                  Country 97.90547
## 92                Saint Lucia                  Country 97.90209
## 93                   Barbados                  Country 97.88090
## 94                  Mauritius                  Country 97.76093
## 95                  Greenland                  Country 97.75769
## 96               Turkmenistan                  Country 97.64197
## 97                     Kuwait                  Country 97.63782
## 98                    Jamaica                  Country 97.57385
## 99         Dominican Republic                  Country 97.41686
## 100                   Albania                  Country 97.30689
## 101                     Qatar                  Country 97.26739
## 102      United Arab Emirates                  Country 97.13076
## 103                      Oman                  Country 97.03222
## 104        Russian Federation                  Country 97.03115
## 105              Saudi Arabia                  Country 96.96500
## 106                   Armenia                  Country 96.88999
## 107                 Venezuela                  Country 96.87538
## 108                     Syria                  Country 96.64943
## 109                   Bahrain                  Country 96.63952
## 110            American Samoa                  Country 96.58036
## 111                    Belize                  Country 96.56692
## 112                     Egypt                  Country 96.51796
## 113                   Lebanon                  Country 96.46144
## 114                Palestine                  Country 96.37350
```

```
## 115                          Morocco                  Country 96.32884
## 116         Northern Mariana Islands                  Country 96.27993
## 117                            Libya                  Country 96.21758
## 118                             Guam                  Country 96.15187
## 119                            Tonga                  Country 96.14428
## 120                       Tajikistan                  Country 96.05603
## 121                          Algeria                  Country 96.03327
## 122   Federated States of Micronesia                  Country 95.92559
## 123                          Tunisia                  Country 95.91315
## 124                             Iran                  Country 95.89016
## 125                        Nicaragua                  Country 95.85928
## 126                             Iraq                  Country 95.85741
## 127                 Marshall Islands                  Country 95.84155
## 128                             Fiji                  Country 95.76432
## 129                      El Salvador                  Country 95.76380
## 130                            Samoa                  Country 95.76228
## 131 Saint Vincent and the Grenadines                  Country 95.71629
## 132                          Vanuatu                  Country 95.69464
## 133                          Estonia                  Country 95.62623
## 134                  Solomon Islands                  Country 95.61161
## 135                         Suriname                  Country 95.34810
## 136                             Peru                  Country 95.28146
## 137                           Guyana                  Country 95.27401
## 138                Equatorial Guinea                  Country 95.17824
## 139                         Kiribati                  Country 95.00594
## 140                 Papua New Guinea                  Country 94.89874
## 141                          Ecuador                  Country 94.87318
## 142                            Gabon                  Country 94.63134
## 143                          Ukraine                  Country 94.48456
## 144              Antigua and Barbuda                  Country 94.00753
## 145                        Sri Lanka                  Country 93.54921
## 146                            Sudan                  Country 93.53246
## 147                           Taiwan                  Country 93.39876
## 148                           Bhutan                  Country 92.85331
## 149                      Switzerland                  Country 92.63828
## 150                         Botswana                  Country 92.41345
## 151                         Honduras                  Country 92.41233
## 152                         Thailand                  Country 92.36613
## 153                            India                  Country 92.31184
## 154                         Dominica                  Country 92.11439
## 155                           Turkey                  Country 92.04578
## 156                          Vietnam                  Country 91.79236
## 157                            Congo                  Country 91.59420
## 158                           Jordan                  Country 91.55416
## 159                            Yemen                  Country 91.53292
## 160                            China                  Country 91.51940
## 161                       Seychelles                  Country 91.48093
## 162                         Pakistan                  Country 91.45064
## 163                       Cape Verde                  Country 91.35201
## 164                       Bangladesh                  Country 91.19101
## 165                          Namibia                  Country 90.95483
## 166                            Ghana                  Country 90.92936
## 167                            Haiti                  Country 90.49722
## 168                         Maldives                  Country 90.35650
```

```
## 169                         Malaysia                  Country 90.21110
## 170                            Kenya                  Country 89.84286
## 171            Sao Tome and Principe                  Country 89.72025
## 172                           Mexico                  Country 89.66448
## 173                            Nepal                  Country 89.61565
## 174                           Uganda                  Country 89.43847
## 175                       Mauritania                  Country 89.36026
## 176                         Cameroon                  Country 89.31353
## 177                           Angola                  Country 89.16236
## 178                    Cote d'Ivoire                  Country 89.07287
## 179                        Guatemala                  Country 88.96181
## 180                           Rwanda                  Country 88.56727
## 181                        Swaziland                  Country 88.32760
## 182                           Zambia                  Country 87.59568
## 183                          Bolivia                  Country 87.29250
## 184                     South Africa                  Country 87.23564
## 185                           Brazil                  Country 87.10227
## 186                             Togo                  Country 86.92593
## 187                      Afghanistan                  Country 86.20671
## 188                          Comoros                  Country 86.12101
## 189                         Djibouti                  Country 85.88850
## 190                       Madagascar                  Country 85.81094
## 191                          Liberia                  Country 85.58770
## 192                           Malawi                  Country 85.32916
## 193                        Indonesia                  Country 85.21461
## 194                         Tanzania                  Country 84.89089
## 195                          Lesotho                  Country 84.80394
## 196                    Guinea-Bissau                  Country 84.45294
## 197 Democratic Republic of the Congo                  Country 83.65864
## 198                       The Gambia                  Country 83.60179
## 199                          Nigeria                  Country 82.95918
## 200                          Eritrea                  Country 82.83619
## 201                         Paraguay                  Country 82.71399
## 202                          Burundi                  Country 82.69759
## 203                      Timor-Leste                  Country 82.25290
## 204                          Senegal                  Country 82.11638
## 205                         Cambodia                  Country 81.64107
## 206                         Zimbabwe                  Country 81.13136
## 207                      South Sudan                  Country 80.80632
## 208                             Laos                  Country 80.48815
## 209                      Philippines                  Country 79.89827
## 210                            Benin                  Country 79.76445
## 211                          Myanmar                  Country 79.70363
## 212         Central African Republic                  Country 77.02683
## 213                      North Korea                  Country 76.79888
## 214                           Guinea                  Country 76.79844
## 215                       Mozambique                  Country 75.70680
## 216                             Chad                  Country 75.49664
## 217                     Sierra Leone                  Country 75.27606
## 218                             Mali                  Country 74.44304
## 219                         Ethiopia                  Country 73.00839
## 220                     Burkina Faso                  Country 72.59247
## 221                            Niger                  Country 67.62856
## 222                          Somalia                  Country 59.05250
```

```
#         group_map(~ qci_table(.x)) %>%
#         data.frame(.) %>%
#         gt() %>%
#         fmt_markdown(columns = TRUE)
```

Computing gender disparity ratio (GDR):

```
gdr = merge(main_data, iso3, by = "location_name") %>%
        select(location_name, year, sex_name, pca_score, type, iso3_countries) %>%
        filter(sex_name %in% c("Male", "Female")) %>%
        distinct() %>%
        spread(sex_name, pca_score) %>%
        mutate(gdr = Female/Male) %>%
        mutate(gdr_cat = ifelse(gdr < 0.95, "low",
                                ifelse(gdr > 1.05, "high", "equal"))
               )
```

Comparing GDR categories in 1990 and 2019:

```
kable(merge(gdr %>%
              filter(year == 1990 & type == "Country") %>%
              group_by(gdr_cat) %>%
              summarise(n = n()),
      gdr %>%
              filter(year == 2019 & type == "Country") %>%
              group_by(gdr_cat) %>%
              summarise(n = n()),
      by = "gdr_cat") %>% setNames(c("gdr_cat", "1990", "2019")))
```

gdr_cat	1990	2019
equal	123	163
high	29	9
low	21	1

Comparing SDI world regions in terms of equal GDR:

```
kable(merge(gdr %>%
              filter(year == 1990 & type == "SDI") %>%
              group_by(location_name) %>%
              summarise(equal = ifelse(gdr_cat == "equal", TRUE, FALSE)),
      gdr %>%
              filter(year == 2019 & type == "SDI") %>%
              group_by(location_name) %>%
              summarise(equal = ifelse(gdr_cat == "equal", TRUE, FALSE)),
      by = "location_name") %>% setNames(c("location_name", "1990", "2019")))
```

location_name	1990	2019
High SDI	TRUE	TRUE
High-middle SDI	FALSE	TRUE
Low SDI	TRUE	TRUE
Low-middle SDI	FALSE	TRUE
Middle SDI	FALSE	TRUE

Comparing World Bank Income Levels in terms of equal GDR:

```r
kable(merge(gdr %>%
              filter(year == 1990 & type == "World Bank Income Levels") %>%
              group_by(location_name) %>%
              summarise(equal = ifelse(gdr_cat == "equal", TRUE, FALSE)),
      gdr %>%
              filter(year == 2019 & type == "World Bank Income Levels") %>%
              group_by(location_name) %>%
              summarise(equal = ifelse(gdr_cat == "equal", TRUE, FALSE)),
      by = "location_name") %>% setNames(c("location_name", "1990", "2019")))
```

location_name	1990	2019
World Bank High Income	TRUE	TRUE
World Bank Low Income	TRUE	TRUE
World Bank Lower Middle Income	FALSE	TRUE
World Bank Upper Middle Income	FALSE	TRUE

Loading required packages

```r
pacman::p_load(clipr,
               tidyr,
               dplyr,
               readstata13,
               data.table,
               FactoMineR,
               foreign,
               RColorBrewer,
               rgdal,
               ggplot2,
               readxl,
               randomcoloR,
               viridis,
               ineq,
               sp,
               LSD,
               reshape,
               ff,
               ffbase)
```

```
## Warning: package 'rgdal' is not available for this version of R
##
## A version of this package for your version of R might be available elsewhere,
## see the ideas at
## https://cran.r-project.org/doc/manuals/r-patched/R-admin.html#Installing-packages

## Warning in p_install(package, character.only = TRUE, ...):

## Warning in library(package, lib.loc = lib.loc, character.only = TRUE,
## logical.return = TRUE, : there is no package called 'rgdal'

## Warning: package 'ffbase' is not available for this version of R
##
## A version of this package for your version of R might be available elsewhere,
```

```
## see the ideas at
## https://cran.r-project.org/doc/manuals/r-patched/R-admin.html#Installing-packages

## Warning in p_install(package, character.only = TRUE, ...):

## Warning in library(package, lib.loc = lib.loc, character.only = TRUE,
## logical.return = TRUE, : there is no package called 'ffbase'

## Warning in pacman::p_load(clipr, tidyr, dplyr, readstata13, data.table, : Failed to install/load:
## rgdal, ffbase
```

The datasets are now created. Next, we w Map

```r
#install.packages(c("cowplot", "googleway", "ggplot2", "ggrepel",
#                    "ggspatial", "libwgeom", "sf", "rnaturalearth", #"rnaturalearthdata", "rgeos", "hav

library("haven")
library("readstata13")
library("dplyr")
library("tidyr")
library("ggplot2")
theme_set(theme_bw())
library("sf")
```

```
## Linking to GEOS 3.11.0, GDAL 3.5.3, PROJ 9.1.0; sf_use_s2() is TRUE
```

```r
library("rnaturalearth")
library("rnaturalearthdata")
```

```
##
## Attaching package: 'rnaturalearthdata'

## The following object is masked from 'package:rnaturalearth':
##
##     countries110
```

```r
library("rgeos")
```

```
## rgeos version: 0.6-4, (SVN revision 699)
##  GEOS runtime version: 3.12.1-CAPI-1.18.1
##  Please note that rgeos will be retired during October 2023,
## plan transition to sf or terra functions using GEOS at your earliest convenience.
## See https://r-spatial.org/r/2023/05/15/evolution4.html for details.
##  GEOS using OverlayNG
##  Linking to sp version: 2.1-3
##  Polygon checking: TRUE

##
## Attaching package: 'rgeos'

## The following object is masked from 'package:bit':
##
##     symdiff

## The following object is masked from 'package:dplyr':
##
##     symdiff
```

```r
map_data = merge(main_data, iso3, by = 'location_id')
map_data$Location = map_data$location_name
map_data$QCI = map_data$pca
```

```
map_data$iso = map_data$iso3_countries
data2 = filter(map_data, year %in% c(1990, 2019), sex_name == 'Both', age_name == "Age-standardized", ty


quantile_values = quantile(data2$QCI, probs = seq(0, 1, 1/5))
data2$quantile = cut(data2$QCI, breaks = quantile_values, include.lowest = TRUE)

world = ne_countries(scale = "medium", returnclass = "sf")
world$iso = world$iso_a3
world = merge(world, data2, by = 'iso')


ggplot(data = world) +
        geom_sf(aes(fill = quantile)) +
        scale_fill_manual(values = c("#a40227", "#f56c42", "#dfbf72", "#a7da68", "#1a9850"), name = "Ag
        theme_void() +
        facet_wrap(~ year, ncol = 1) +
        theme(legend.key.size = unit(0.4, 'cm'))
```

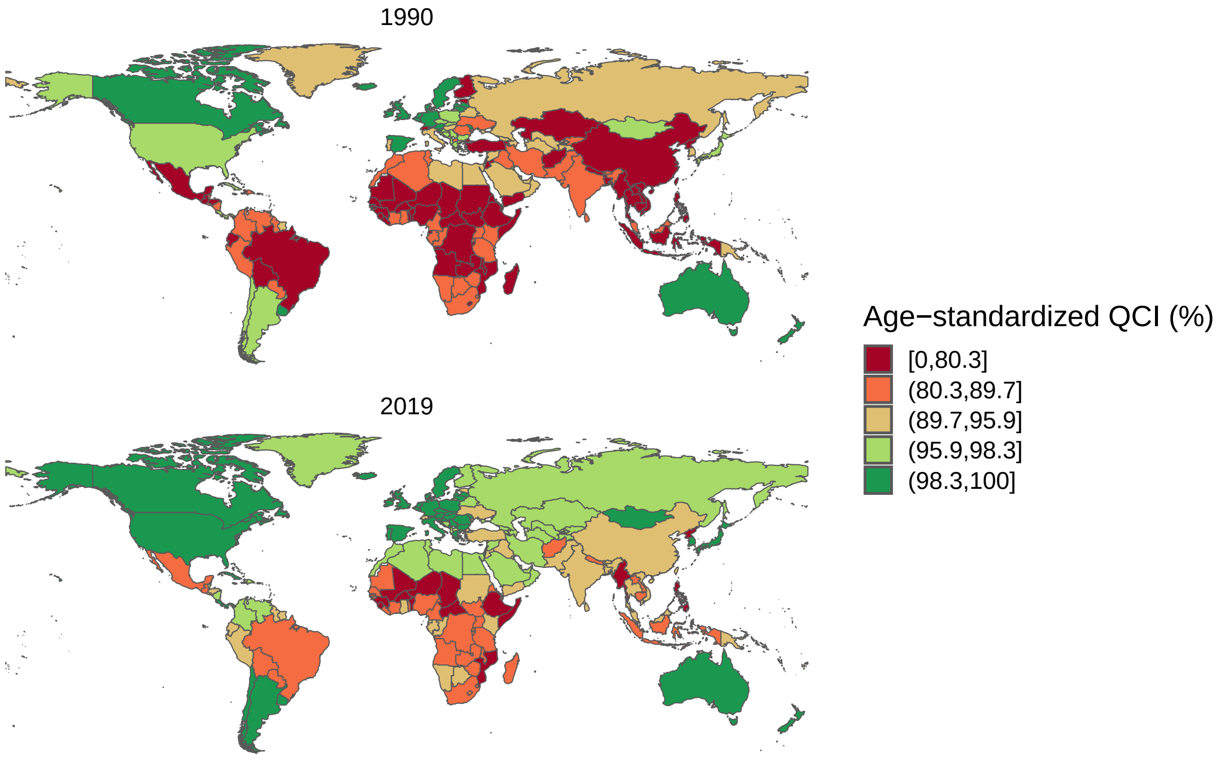


```
# dev.copy(tiff, "figures/Figure3.tiff", width = 20, height = 10, units = "cm", res = 300)
# dev.off()
```

GDR

```
# install.packages(c("cowplot", "googleway", "ggplot2", "ggrepel",
#                    "ggspatial", "libwgeom", "sf", "rnaturalearth", "rnaturalearthdata", "rgeos", "have

library("haven")
library("readstata13")
library("dplyr")
```

```
library("tidyr")
library("ggplot2")
theme_set(theme_bw())
library("sf")
library("rnaturalearth")
library("rnaturalearthdata")
library("rgeos")
gdr_data = merge(main_data, iso3, by = 'location_id')
gdr_data$Location = gdr_data$location_id
gdr_data$QCI = gdr_data$pca
gdr_data$iso = gdr_data$iso3_countries
data2 = filter(gdr_data, year %in% c(1990, 2019), sex_name %in% c('Male', 'Female'), age_name == "Age-s
data3 = select(data2, location_id, iso, sex_name, year, QCI)
data4 = spread(data3, sex_name, QCI)
data4$GDR = data4$Female/data4$Male

breaks <- c(0, 0.5, 0.95, 1.05, 2.0, Inf)
labels <- c("< 0.5", "[0.5, 0.95)", "[0.95, 1.05]", "(1.05, 2.0]", "> 2.0")
data4$quantile <- cut(data4$GDR, breaks = breaks, labels = labels, include.lowest = TRUE)

world = ne_countries(scale = "medium", returnclass = "sf")
world$iso = world$iso_a3
world = merge(world, data4, by = 'iso')

ggplot(data = world) +
        geom_sf(aes(fill = quantile)) +
        scale_fill_manual(values = c("#878787", "#b7b7b7", "#64c2a5", "#b1abd0", "#7f74ac"), name = "Ag
        theme_void() +
        facet_wrap(~ year, ncol = 1) +
        theme(legend.key.size = unit(0.4, 'cm'))
```

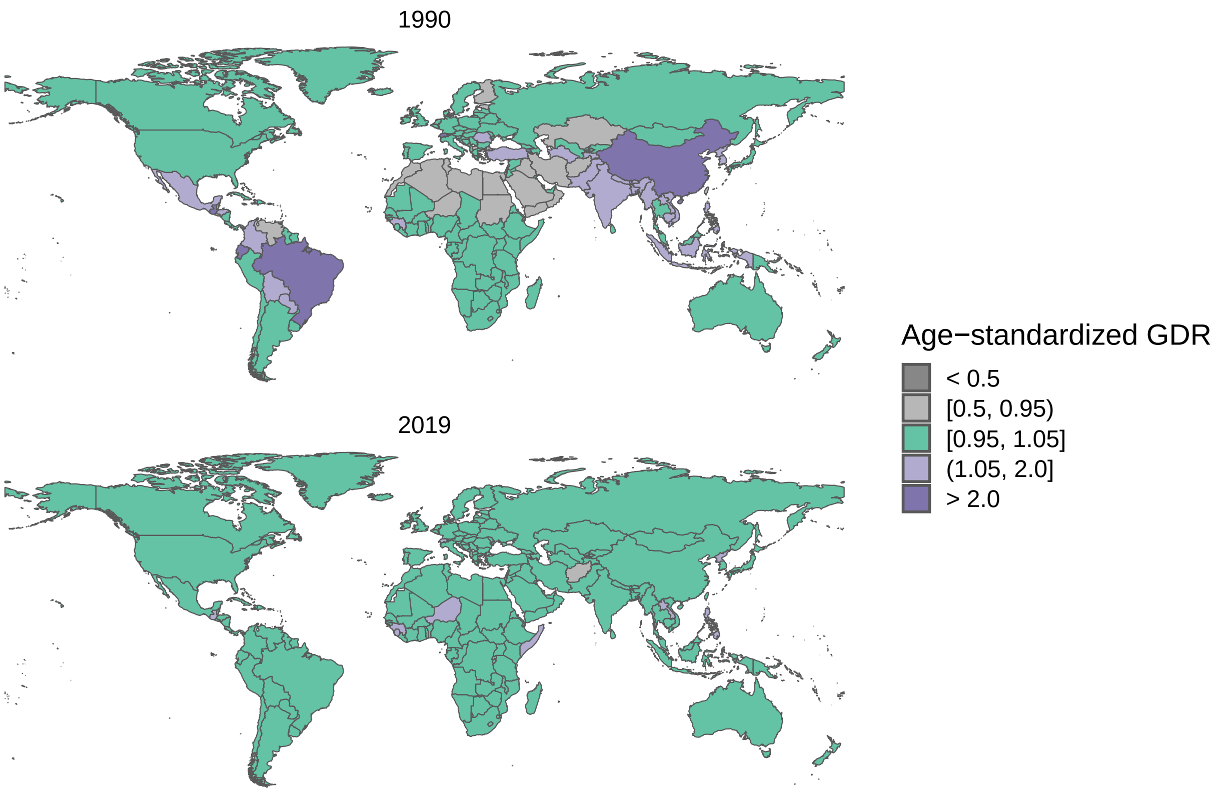


```r
# dev.copy(tiff, "figures/Figure2.tiff", width = 20, height = 10, units = "cm", res = 300)

dev.off()
```

```
## null device
##           1
```

```r
data3 = select(data2, Location, QCI)
```
