# Supplementary material for: Global, regional, and national survey on burden and Quality of Care Index (QCI) of orofacial clefts: Global burden of disease systematic analysis 1990–2019
Source: PLoS One. 2025 Jan 8;20(1):e0317267. doi: 10.1371/journal.pone.0317267 (PMC11709310; doi:10.1371/journal.pone.0317267)
Supplement: Attachment — Submitted filename: rebuttal_letter.docx [file pone.0317267.s002.docx]

Associate Editor:

The manuscript suffers from grammatical errors throughout

The introduction is poorly structured, it darts around between incomplete lists of cleft configurations and classifications.

The authors state orofacial clefts ‘roughly’ affect 1 in 700 births, for a manuscript claiming to be ‘global, regional and national’ in its scope, this figure is a gross oversimplification.

The authors state that children with clefts require orthodontics and dentistry, it would appear that orthodontics, according to this statement, is not a branch of dentistry.

‘This comprehensive care constitutes only a portion of each discipline’s clinical workload’ this is just one of the examples of how the manuscript is unnecessarily padded out.

The authors refer to a ‘controversy’ in management of clefts due to the involvement of various disciplines, this is a statement without a reference and is potentially irresponsible to be included in scientific literature.

The authors have apprently created a ‘novel’ ‘QCI’ and proceed to list its qualities in the introduction without introducing the reader to its nuances.

Perhaps the authors could find a synonym for the word ‘novel’ as it seems to have been used repeatedly in quick succession, almost appearing to thrust upon the reader the claim that the authors have done something ‘novel’.

The authors have claimed that their ‘innovative’ approach has bridged the ‘current data gap’ to facilitate ‘deeper comprehension of discrepancies’ among ‘various nations and regions. These statements again, just pad out the manuscript and provide no insight into what the authors have actually done.

No research gap has as such been stated clearly, the solution to this perceived gap and the approach taken by the authors has been replaced in the introduction by terms and statements which do not convey scientific information and merely serve as embellishments.

Authors’ response: Thanks for your comments. We completely modified the Introduction to address all your comments.

The authors state that the protocol was ‘meticulously’ published. Perhaps a senior author would want to read the manuscript to advise the others if making such statements is appropriate. While this is being done, it would be appropriate if a senior author actually involved in delivering cleft care were involved as well.

Acronyms, in certain places, have been repeatedly presented with their expansions and in some other places, have been completely ignored.

Authors’ response: Thanks for your comment. We amended the manuscript accordingly.

Stating that high and middle income countries are more likely to adopt a receptive stance towards cost effective and sustainable interventions is irresponsible and potentially controversial. One could argue that this is condescending.

‘It is imperative for them to consider these factors during the policymaking process’ it appears the authors are addressing a certain crowd through this manuscript without explicitly stating so.

Stating that a high income country which started off with a good score had ‘worsened’ in its cleft care approach would seem a little unfair.

The manuscript discusses African countries as ones with the worse scores in cleft care, with certain European nations being named as the best.

One could argue as to the validity of the ‘global’ reach of this study where Asian and South American countries are more or less excluded from the discussion.

Authors’ response: Thanks for your comment. We fully modified the Discussion section based on your comments.

Reviewer #1: This is an interesting topic and the ability to be able to assess the quality of care for orofacial clefts on a global scale is certainly appealing. The authors demonstrate good scientific rigour to pre-publish their protocol and have their code and data freely accessible. The authors clearly have had prior experience of handling these indices and this data set, so would be well placed to do a study of this sort. However, I do have concerns about the actual relevance of these indices to orofacial clefts. Even though they may be relevant to other health conditions, I am not sure they are relevant for a birth anomaly such as orofacial. Wang et al 2023 (<https://www.ncbi.nlm.nih.gov/pmc/articles/PMC10228319/#:~:text=Globally%2C%20the%20DALYs%20of%20orofacial,72%25%20to%20%E2%88%9238%25>) published an article reporting orofacial cleft DALYs from GBD data from the same time frame. I am not convinced that this current study adds anything useful in addition to this recent publication.

Authors’ response: Thanks for your comment. We agree that our results may share points with the results from Wang et al.; however, in our manuscript, we introduced a more comprehensive yet easy-to-interpret scale (i.e., QCI). QCI is easy to comprehend since it provides a number between 0-100. Therefore, we can be more confident in the ranking of the countries and their quality of care. Because, unlike DALY (which is heavily affected by many factors and should be used alongside other metrics), we used all the other available measures to form a robust measure to quantify the quality of care.

Abstract
It is difficult to understand from the abstract why this study was done and what exactly has been done. Specifically, it is not clear what QCI is and what it measures.
There are too many abbreviations defined in the abstract that are not then used. Suggest restricting to just QCI as the sole abbreviation used in the abstract

Authors’ response: Thanks for your comment. We modified the Abstract accordingly.

Introduction
The first paragraph is vague and repetitive. This article is not an overview of the cleft phenotype. It would be better to have more focused and therefore relevant discussion of the topic of this article.

The second paragraph is also vague and makes statements that if read alone could be interpreted incorrectly. For example – ‘research findings have indicated a heightened occurrence of structural brain abnormalities’ references a single study of 74 children from the USA. This is a strong statement about a complex association that could have negative repercussion for patients and families and is misleading as a throw away comment in a single sentence.

The third paragraph states that comprehensive care only constitutes a proportion of each discipline’s clinical workload. This may be true for many countries but not all. For example, in the UK, centralised cleft teams include health professionals who are solely involved in the provision of comprehensive cleft care.

The fourth paragraph finally addresses the focus of the article but it does not describe The Global Burden of Disease study for the uninitiated and there are no references provided. It would be useful for the reader to understand the fundamentals of this study as the source of data in this article. Reference 14, given later in the methods section, would be more useful in the introduction

The abbreviation QCI is used in the introduction without prior explanation in full words

Overall, the introduction is inadequate. This article is about an assessment of the effectiveness of cleft care. Assessing effectiveness and outcomes (of which DALYs and QCIs could arguably be classified) in cleft is notoriously complex due to the multidisciplinary nature of the condition with multiple interventions at different time points. It would have been much more valuable to have explored the complexities of outcome reporting in cleft and how this ‘novel’ method may fit and be useful amongst the more established ones.

Authors’ response: Thanks for your comments. We thoroughly edited the Introduction and addressed all your comments.

Methods
How can a protocol be meticulously published? Suggest removal of word ‘meticulously’.

Authors’ response: Thanks for your comments. We removed that word.

The four distinct indices are explained but I am concerned about the relevance and applicability to cleft and this is not discussed in the methods section as to whether this was considered.
• Prevalence to incidence ratio – this may be relevant to a disease process that can be cured (ie bipolar disorder which is the focus of a QCI paper recently published <https://pubmed.ncbi.nlm.nih.gov/37353952/>) but I question its relevance for a congenital anomaly that is present for life and does not have a cure. What does this ratio actually mean for a person born with cleft?

Authors’ response: Thanks for your comments. Your argument is valid. However, a quick look at the data sources of the GBD study (https://ghdx.healthdata.org/search/site/orofacial%20cleft) shows that the included studies considered that orofacial clefts can be “treated” just like other disorders. Therefore, “prevalence” is also relevant in this context. For example, this is one of the included studies: https://ghdx.healthdata.org/record/epidemiological-study-orofacial-clefts-among-population-eastern-slovakia-during-period-1996.

• Mortality to incidence ratio – how can this serve as an indicator for effectiveness in cleft care provision? In resource poor settings, mortality may be associated with malnutrition in a child born with a cleft palate but as an overall indicator of the effectives of care outcomes in orofacial cleft, I suspect that this is a poor measure.

Authors’ response: Thanks for your comments. Just as you mentioned, malnutrition is common in patients with orofacial cleft and can be a cause of death (https://ghdx.healthdata.org/record/epidemiological-study-orofacial-clefts-among-population-eastern-slovakia-during-period-1996). Our reason for including this index was to have a comprehensive index that not only considers care for the disease but also broader care needs for orofacial cleft patients (like their special dietary needs).

• DALYs to prevalence ratio seems more intuitively relevant to cleft
• YLLs to YLDs could also be relevant for cleft and looks specifically at compromised survival status, rather than claiming to be a measure of care provision overall
The overall question here: is the multivariate index QCI a useful measure in cleft? What is the validity of it and how has this been tested? In my opinion the name “quality care index’ is misleading because this index does not measure the quality of care in cleft.

Authors’ response: Thanks for your comments. We chose the name “quality of care index” since it is an established name in the studies using this methodology. The validity of this index is mentioned in the last section of the Methods section (under the “Validation” subheading). While we acknowledge the shortcomings of this methodology (for all diseases, not just for orofacial clefts), we still believe this can serve as a measure to quantify the quality of care. At least it is the best that we can do.

It is not clear whether analysis was done using data from 2 times points only (1990 and 2019) or whether data was used from each of the 29 years. If only two timepoints was used, I am not sure this is very representative of the changes that could have occurred over the time period. Did the authors consider the use of time series analysis to capture the rich data across the 29 year time frame?

Authors’ response: Thanks for your comments. We have the data for all 29 years (available on our OSF repository: <https://osf.io/u7v8e>). However, we could not present all of our data in the paper. We also showed the necessary data for all the years in Figure 1 (temporal trend for both genders and each separately for all the years.

Results
DALY scores seem understandable

QCI less so. How can the scores in 1990 be at the extremes of the possible QCI scale with one country (Norway) having 100 and another (Brazil) have 0 – are these useful values? How useful is a measure where one country (Norway) is at the top of the scale at the start of the time period and then is judged to have decreased overtime (when this is actually only by 0.5) when it would have been impossible to improve its QCI?

Authors’ response: Thanks for your comment. The backbone of QCI is PCA. As mentioned in the Methods, “The first-ranked component of the PCA, which is a linear combination of all variables, captures the majority of the variables' information and was considered as the Quality-of-Care Index (QCI).” This means based on the four indices we introduced, PCA analysis captures the largest variation in the data can be easily identified. It provides five different components. The first one always includes the largest information in terms of the rankings. We captured this and transformed it to a value between 0-100 which is QCI.

While 100 to 99.9 seems to be decreasing, we mentioned in the results that “Norway maintained the highest QCI score at 99.9”.

Table 1:
In the last column heading ‘QCI change 1990 to 2019) – what is the value being measured here? Abbreviations in the table should be explained so that the table can be understood in isolation

Authors’ response: Thanks for your comment. We subtracted the 2019 QCI from 1990 QCI (change). We added explanations to Table 1.

Figure 1
Good figure – shows the QCI data points for each year but were these 29 data points actually used in the analysis or just the two points at 1990 and 2019?

Authors’ response: Thanks for your comment. All the points were analyzed. However, since showing other points in the manuscript seemed redundant due to being uninformative, we sticked to the years 1990 and 2019 for other parts of the results.

Figure 2
Good figure

Authors’ response: Thanks.

Figure 3:
Unintelligible. Which year are the QCIs relating to?

Authors’ response: Thanks for your comment. We added 2019 to the caption.

Figure 4:
Visually a good figure but applicability of the QCI as a useful index in cleft care questionable

Authors’ response: Thanks for your comment. We added explanations above.

Discussion
The first paragraph is generalised and vague and do not focus on the specific findings in this study.

Authors’ response: Thanks for your comment. We changed the order of the paragraphs in the discussion.

The second paragraph discusses the reason for using prevalence and is therefore more suited to the methods section

Authors’ response: Thanks for your comment. We transferred the paragraph to the Methods section.

The third and fourth paragraph do address the findings of this study

Authors’ response: Thanks for your comment. We changed the order of the paragraphs in the discussion.

The fifth paragraph dives off again into generalised cleft information. How can speech and hearing difficulty contribute to mortality? Malnutrition and infection can lead to mortality. How is telemedicine relevant to the argument?

Authors’ response: Thanks for your comment. We added additional information to the Discussion and amended it accordingly.

Overall the discussion lacks a clear structure and is difficult to follow

Authors’ response: Thanks for your comment. We substantially changed the Discussion.

Conclusions
The conclusions recommend regions with falling QCI to adopt a more proactive approach to addressing orofacial cleft care in future. Not only is this recommendation vague without a clear and bespoke plan, but it is also a significant leap of faith from the findings of this study, which is at best not useful and at worst it could be interpreted as condescending.

Authors’ response: Thanks for your comment. We removed the mentioned parts from the Conclusion.

Reviewer #2: i think that the value of this study is in identifying the countries at the lower end of the scale. Mortality is rarely associated with simple orofacial clefting in higher-income countries. This should be emphasized in the manuscript. Later in the manuscript, I think it is over simplistic to suggest that lower-middle income countries should take a more proactive approach to management of orofacial clefting. There are multiple levels of challenge in LMICs that preclude advanced multidiscipliinary care of infants born with orofacial clefts. A more in depth exploration of these issues should be included in the paper.

Authors’ response: Thanks for your comment. We addressed all your comments alongside comments by other reviewers.
